# Distinct Roles of m^5^C RNA Methyltransferase NSUN2 in Major Gynecologic Cancers

**DOI:** 10.3389/fonc.2022.786266

**Published:** 2022-02-25

**Authors:** Lingfang Wang, Jian Zhang, Yingfeng Su, Yasen Maimaitiyiming, Siqi Yang, Zhangjin Shen, Shitong Lin, Shizhen Shen, Guankai Zhan, Fenfen Wang, Chih-Hung Hsu, Xiaodong Cheng

**Affiliations:** ^1^Zhejiang Provincial Key Laboratory of Precision Diagnosis and Therapy for Major Gynecological Diseases, Women’s Hospital, Zhejiang University School of Medicine, Hangzhou, China; ^2^Women’s Hospital, Institute of Genetics and Department of Environmental Medicine, Zhejiang University School of Medicine, Hangzhou, China; ^3^Department of Hematology of First Affiliated Hospital and Department of Public Health, Zhejiang University School of Medicine, Hangzhou, China; ^4^Department of Clinical Research Center, Women’s Hospital, Zhejiang University School of Medicine, Hangzhou, China; ^5^Cancer Biology Research Center (Key Laboratory of the Ministry of Education), Department of Obstetrics and Gynecology, Tongji Hospital, Tongji Medical College, Huazhong University of Science and Technology, Wuhan, China

**Keywords:** NSUN2, m^5^C, cervical cancer, ovarian cancer, endometrial cancer, YBX1, KRT13

## Abstract

RNA methylation has recently emerged as an important category of epigenetic modifications, which plays diverse physiopathological roles in various cancers. Recent studies have confirmed the presence of 5-methylcytosine (m^5^C) modification on mammalian mRNAs, mainly modified by NOP2/Sun RNA methyltransferase family member 2 (NSUN2), but little is known about the underlying functions of m^5^C. Gynecologic cancers are malignancies starting from women’s reproductive organs. The prevalence of gynecologic cancers leads to a massive economic burden and public health concern. In this study, we investigated the potential biological functions of NSUN2 in common gynecologic cancers including cervical cancer, ovarian cancer, and endometrial cancer. Remarkably, distinct scenarios were found. The levels of NSUN2 did not show alteration in endometrial cancer, and in ovarian cancer, depletion of upregulated NSUN2 did not reduce carcinogenesis in cancer cells, suggesting that the upregulated NSUN2 might be an incidental effect. On the contrary, NSUN2 played a role in tumorigenesis of cervical cancer; depletion of upregulated NSUN2 notably inhibited migration and invasion of cancer cells, and only wild-type but not catalytically inactive NSUN2 rescued these malignant phenotypes of cancer cells. Mechanistically, NSUN2 promoted migration and invasion by leading to m^5^C methylation on keratin 13 (*KRT13*) transcripts, and methylated *KRT13* transcripts would be recognized and stabilized by an m^5^C reader, Y-box binding protein 1 (YBX1). Collectively, these results not only displayed the nature of diversity among human malignancies, but also demonstrated a novel NSUN2-dependent m^5^C-YBX1-KRT13 oncogenic regulatory pathway.

## Introduction

Due to the single-stranded nature and relatively flexible structure, RNA carries a diverse array of post-transcriptional chemical modifications (over 160), including N^6^-methyladenosine (m^6^A), N^1^-methyladenosine (m^1^A), 5-methylcytosine (m^5^C), 5-hydroxymethylcytosine (hm^5^C), and 2′-O-methylation (Nm) (1). Among these, m^6^A and m^5^C are the top two common modifications existing on mammalian mRNA (2). Recent studies have well unveiled the functions of m^6^A modification in numerous biological processes (3–5), but little is known about the functions of m^5^C modification. m^5^C is mainly catalyzed by the NOL1/NOP2/SUN domain (NSUN) family proteins containing seven members (NSUN1–7) in humans, and the DNA methyltransferase homologue DNMT2 (6). Among these, NSUN2 is the most well-characterized m^5^C methyltransferase with robust catalytic activity for mRNA methylation (7). It is reported that two residues, named releasing (cysteine 271) and catalytic (cysteine 321) sites, play key roles in the enzymatic activity of NSUN2. To be specific, cysteine 321 of NSUN2 (C321) is essential for the formation of a covalent bond between NSUN2 and the cytosine pyrimidine ring, and the second conserved cysteine at position 271 (C271) is essential for the release of the methylated RNA (8, 9). NSUN2-mediated regulation of certain mRNAs has been reported to take part in multiple biological processes, such as stress response (10), regulation of mitotic spindle stability (11), and cell proliferation (12). In addition, several studies have reported high expression of NSUN2 in certain types of neoplasms including breast cancer, colorectal cancer, and lung cancer (13, 14). However, little is known about how aberrantly expressed NSUN2 contributes to pathogenesis and development of cancer.

Gynecologic cancers, a group of cancers that affect the female reproductive system, are a major threat to women’s health, including cervical cancer, ovarian cancer, uterine cancer, vaginal cancer, and vulvar cancer (15). Among these, cervical, ovarian, and endometrial carcinoma are the top three common subtypes accounting for 95% of gynecologic cancers (16–18). Specifically, endometrial cancer is the most common gynecologic malignancy and the fourth most common malignancy affecting women in the United States, leading to over 11,000 deaths of women per year (2018) (19). It is reported that more than half of endometrial cancer are attributable to obesity (20, 21). Unfortunately, as a result of the increasing worldwide obesity rate, the incidence and mortality of endometrial cancer continue to grow (21). A multivariate linear regression model predicted that the incidence of endometrial cancer will reach 42.13 cases per 100,000 women in 2030, a 55% increase over 2010 endometrial cancer incidence (21). Cervical cancer is the fourth most commonly diagnosed cancer and the fourth leading cause of cancer death in women across the globe, with an estimated 604,127 new cases and 341,831 deaths in 2020 (18). Although cervical cancer is considered preventable by using human papillomavirus (HPV) vaccine and secondary prevention measures, less than 30% of low- and middle-income countries have implemented national HPV vaccination programs and only 44% of women in low- and middle-income countries have ever been screened for cervical cancer (18). Ovarian cancer is the leading cause of death from female gynecological cancers and claims the lives of approximately 150,000 women every year worldwide (22). A lack of accurate screening tools and vague symptoms result in 75% of ovarian cancer patients being diagnosed with advanced disease (23). The standard treatment for advanced ovarian cancer is primary surgical cytoreduction combined with platinum-based chemotherapy. However, recurrence and resistance of ovarian cancer to chemotherapy is yet to be resolved in clinic (24). Collectively, there is an urgent need to reveal unknown aspects of gynecological cancer pathogenesis and progression, so as to provide new approaches for the prevention and treatment of gynecolgical cancers.

To understand the potential roles of RNA m^5^C modification in tumorigenesis of major gynecological cancers, we investigated the biological functions of NSUN2 in cervical, ovarian, and endometrial carcinoma, and intriguingly found that the roles of NSUN2 seem quite different among these subtypes of gynecological cancers. In endometrial carcinoma, the expression levels of NSUN2 were similar to that in normal tissue, suggesting that NSUN2 might be dispensable for tumorigenesis in endometrial carcinoma. Although NSUN2 levels were upregulated in both ovarian and cervical cancers, knockdown of NSUN2 did not reduce aggressive phenotypes of ovarian cancer cells, suggesting that upregulation of NSUN2 is likely an accompanied effect but not a fundamental driving force in ovarian cancer. In contrast, NSUN2 played a robust role for tumorigenesis in cervical cancer *via* promoting m^5^C modification on *KRT13* transcripts, which were then recognized and stabilized by an m^5^C reader protein YBX1. As a whole, our findings not only demonstrated distinct roles of NSUN2 in different gynecological cancers, which represented the nature of diversity and complexity of human malignancies, but also discovered a novel NSUN2-dependent m^5^C-YBX1-KRT13 oncogenic regulatory pathway in cervical cancer. This new regulatory mechanism would provide potential biomarker and therapeutic targets for future study and treatment of cervical cancer.

## Materials and Methods

### Bioinformatics Analysis

GEO expression profiling dataset was used to analyze the mRNA expression of *NSUN2* in cervical, ovarian, and endometrial cancer. GEPIA (http://gepia.cancer-pku.cn/) was used to analyze the mRNA levels of NOL1/NOP2/SUN domain (NSUN) family proteins in the major gynecologic cancers. UALCAN website (http://ualcan.path.uab.edu/) was used to analyze the mRNA and protein expression of NSUN2 in cervical, ovarian, and endometrial cancer. cBioPortal website (https://www.cbioportal.org/) was used to explore the RNA alteration of NSUN2 in cervical, ovarian, and endometrial cancer. The overall survival plots related to *NSUN2* and *NSUN6* expression in cervical, ovarian, and endometrial cancer were obtained by Kaplan–Meier plotter (http://kmplot.com/analysis/).

### Clinical Samples

Twenty cervical carcinoma tissues and 20 normal cervical epitheliums, 20 ovarian cancer tissues, and 11 normal ovarian tissues were collected to analyze the mRNA expression of NSUN2 in cervical cancer and ovarian cancer. Five pairs of cervical tumor and adjacent tissue samples and 6 pairs of ovarian tumor and adjacent tissue samples were collected to analyze the protein expression of NSUN2 in cervical and ovarian cancer. We collected 30 paraffin-embedded cervical cancer wax and 29 paraffin-embedded normal cervical tissue, and made a tissue chip to explore the expression of NSUN2 in cervical cancer. All the samples were collected from December 2019 to December 2020 in Women’s hospital, School of Medicine, Zhejiang University, China. Patients provided informed consent to obtain samples, and the study was subjected to approval by the Hospital Ethical Committee. Tissue samples were stored in RNA store solution at 4°C overnight and stored at −80°C until use.

### Plasmids Construction

pHAGE-CMV-NSUN2-FLAG-HA, which expresses FLAG-HA-tagged NSUN2, was constructed by inserting NSUN2 cDNA fragment into pHAGE-CMV-FLAG-HA. shNSUN2 resistant NSUN2 wild-type (NSUN2-WT) and mutant (with point mutation at cysteine 271 and 321) plasmids (NSUN2-DM) were constructed by introducing point mutations through the use of KOD One™ PCR Master Mix -Blue- (KMM-201, TOYOBO) as previously reported (9). Plasmids pHAGE-CMV-KRT13-FLAG-HA, pHAGE-CMV-YBX1-FLAG-HA, and pHAGE-CMV-ALYREF-FLAG-HA, which express FLAG-HA-tagged KRT13, YBX1, and ALYREF, respectively, were constructed by inserting corresponding cDNA fragment after CMV promoter of pHAGE-CMV-FLAG-HA. The KRT13 promoter and GAPDH promoter were cloned from genomic DNA. Plasmid pGL3-KRT13-luciferase was constructed by inserting KRT13 promoter into pGL3-luciferase. Plasmid pGL3-GAPDH-mcherry was constructed by inserting GAPDH promoter into pGL3-mcherry. Plasmids pLKO.1-shNSUN2-1 and pLKO.1-shNSUN2-2, which were designed to suppress endogenous NSUN2, were constructed by inserting shRNA-targeting sequence after U6 promoter of pLKO.1 using KOD One™ PCR Master Mix -Blue-. The sense sequences are listed below:

shNSUN2-1: 5’-GAGCGATGCCTTAGGATATTACTCGAGTAATATCCTAAGGCATCGCTC-3’,

shNSUN2-2: 5’-CAGTGGAAGGTAATGACGAAACTCGAGTTTCGTCATTACCTTCCACTG-3’.

The generation and infection of lentivirus were conducted according to the protocol from Addgene.

### Cell Line Culture and Transfection

The human cervical cancer cell lines SiHa and CaSki, and the human ovarian cancer cell lines A2780 and SKOV3 were purchased from the American Type Culture Collection (ATCC, USA). The SiHa cell line was cultured in DMEM (BI, Israel) containing 10% FBS and maintained at 37°C in 5% CO_2_; CaSki and A2780 cell lines were cultured in RPMI-1640 (BI, Israel) containing 10% FBS. The SKOV3 cell line was cultured in MCCOY’S 5A (M9420, Solarbio) containing 10% FBS. We used PEI MAX^®^–Transfection Grade Linear Polyethylenimine Hydrochloride (49553-93-7, Polysciences) to transfect plasmid and Lipofectamine™ RNAiMAX Transfection Reagent (13778150, Invitrogen) to transfect siRNA according to the manufacturer’s instructions to regulate gene expression. The siRNA sequences are listed below:

SiYBX1-1: 5’-GGACGGCAATGAAGAAGAT-3’

SiYBX1-2: 5’-CCACGCAATTACCAGCAAA-3’

SiALYREF-1: 5’-GCACGATCTTTTCGACAGT-3’

SiALYREF-2: 5’-GAGGTGGCATGACTAGAAA-3’

SiKRT13-1: 5’-CUACUACAAGACCAUUGAATT-3’

SiKRT13-2: 5’-GCCAGAACCAAGAGUACAATT-3’

### RNA Extraction, PCR, and qRT-PCR

The total RNA of cells was extracted with RNAiso Plus (9109, Takara) under the manufacturer’s guidance. The cDNA was synthesized by PrimeScript™ RT Reagent Kit with gDNA Eraser (RRO47A, Takara) according to the manufacturer’s instructions. Light Cycler 480 (Roche) was used to detect the expression levels of target genes using 2X Universal SYBR Green Fast qPCR Mix (RK21203, ABclonal). The examined genes were normalized to GAPDH expression. The specific primer sequences of targeted genes are displayed in **Supplementary Table S1**.

### RNA-seq

The total RNA of cells was extracted with RNAiso Plus (9109, Takara). RNA-seq was performed by Cloudseq Biotech Inc. (Shanghai, China) following the published procedure (25). The detailed information is listed below. Total RNA was used for removing the rRNAs with NEBNext rRNA Depletion Kit (New England Biolabs, Inc., Massachusetts, USA) following the manufacturer’s instructions. RNA libraries were constructed by using NEBNext^®^ Ultra™ II Directional RNA Library Prep Kit (New England Biolabs, Inc., Massachusetts, USA) according to the manufacturer’s instructions. Libraries were controlled for quality and quantified using the BioAnalyzer 2100 system (Agilent Technologies, Inc., USA). Library sequencing was performed on an illumina Hiseq instrument with 150-bp paired-end reads.

### RNA Bisulfite Sequencing

The total RNA of cells was extracted with RNAiso Plus (9109, Takara). RNA bisulfite sequencing and the following bioinformatic analysis were performed by Cloudseq Biotech Inc. (Shanghai, China). Briefly, 2 μg of total RNA for each sample was rRNA depleted using NEBNext^®^ rRNA Depletion Kit (New England Biolabs, Inc., Massachusetts, USA). rRNA-depleted RNA was bisulfite converted and purified using the EZ RNA Methylation Kit (Zymo Research). RNA libraries were then constructed with TruSeq Stranded Total RNA Library Prep Kit (Illumina, San Diego, CA, USA) according to the manufacturer’s instructions. The library quality was evaluated with the BioAnalyzer 2100 system (Agilent Technologies, Inc., USA). Library sequencing was performed on an Illumina Hiseq instrument with 150-bp paired-end reads.

Paired-end reads were harvested from Illumina HiSeq 4000 sequencer and were quality controlled by Q30. After 3’ adaptor-trimming and low-quality reads removing by cutadapt software (v1.9.3), the high-quality trimmed reads (clean reads) were aligned to the human reference genome (UCSC HG19) using meRanGs (one component of meRankTK) software with default parameters. The methylation status of each C within the genome was extracted by meRanCall (one component of meRankTK) software. meRanCompare (one component of meRankTK) software was used to identify differentially methylated sites (DMSs) on mRNA. The DMSs in mRNA were annotated with the Ensembl database to connect DMCs with the gene/transcript annotation. Then, GO and pathway analysis were conducted on those DMC-related genes.

### RNA Binding Protein Immunoprecipitation-qPCR

The transfected CaSki cells were washed twice with ice-cold PBS and lysed in 1 ml of RIP Lysis buffer [150 mM KCl, 25 mM Tris, 5 mM EDTA, 0.5% Triton X-100, 0.5 mM DTT, Protease inhibitor (1:100), and RNase inhibitor (1:1,000)] on ice for 30 min. Cell lysates were centrifuged at 14,000 *g* at 4°C for 10 min to obtain clear lysates. Ten percent supernatant was collected as input, and the remaining supernatant was incubated with primary antibody and protein A/G Magnetic Beads in 900 μl of RIP Lysis buffer on a rotary homogenizer at 4°C for 4 h. Bounded RNAs were immunoprecipitated with beads and the beads were washed four times with RIP buffer (150 mM KCl, 25 mM Tris, 5 mM EDTA, and 0.5% Triton X-100). Then, beads were resuspended with 10 μl of 10% SDS, 10 μl of Proteinase K, and 130 μl of RIP buffer and digested at 55°C for 30 min. RNA in immunoprecipitation or input group was recovered with RNAiso Plus according to the manufacturer’s instruction. The relative enrichment of interest RNA was determined by RT-qPCR. Data were normalized to input.

### m^5^C Methylated RNA Immunoprecipitation

The total RNA of cells was extracted with RNAiso Plus (9109, Takara). The extracted total RNA was randomly fragmented by RNA fragmentation reagents (AM8740, Thermo Scientific). Ten percent volume RNA was used as input, and the remaining RNA was incubated with anti-m^5^C antibody (A18870, ABclonal) and protein A/G Magnetic Beads in 900 μl of RIP Lysis buffer on a rotary homogenizer at 4°C for 4 h. Bounded RNAs were immunoprecipitated with beads and beads were washed four times with RIP buffer (150 mM KCl, 25 mM Tris, 5 mM EDTA, and 0.5% Triton X-100). Then, beads were resuspended with 10 μl of 10% SDS, 10 μl of Proteinase K, and 130 μl of RIP buffer and digested at 55°C for 30 min. RNA in immunoprecipitation or input group was recovered with RNAiso Plus according to the manufacturer’s instruction. The levels of enriched fragmented RNA and input were measured by one-step qPCR using TransScript^®^ II One-Step RT-PCR SuperMix (AH411-02, TransScript). IP enrichment ratio was calculated as the ratio of its amount in IP to that in the input. The specific primer sequences are displayed in **Supplementary Table S1**.

### RNA Stability Assay

Transfected CaSki cells were treated with 10 μg/ml actinomycin D after transfection (48 h). Cells were collected at the indicated time points. The total RNA was extracted and analyzed by RT-qPCR.

### Luciferase Reporter Assay

Before the experiment, the KRT13 promoter and GAPDH promoter were cloned from genomic DNA. Plasmid pGL3-KRT13-luciferase was constructed by inserting KRT13 promoter into pGL3-luciferase. Plasmid pGL3-GAPDH-mcherry was constructed by inserting GAPDH promoter into pGL3-mcherry. Then, YBX1 knockdown and control CaSki cells were co-transfected with pGL3-KRT13-luciferase and pGL3-GAPDH-mcherry. After transfection, the cells were harvested and the total RNA of cells was extracted with RNAiso Plus (9109, Takara). The relative luciferase level was determined by RT-qPCR. Data were normalized to mcherry level.

### Western Blot and Antibody

Cells were lysed on ice in ice-cold lysis buffer (50 mM Tris-HCl, 150 mM NaCl, 5 mM EDTA, 0.1% SDS, 1% Triton X-100, and 5% Glycerol) containing protease inhibitors (B14001, Bimake). Equal amounts (20 μg) of total extract protein was separated on a 4%–20% polyacrylamide gel (M00656, GenScript) and was electrically transferred onto NC membrane. The antibodies used are listed as follows: NSUN2 (20854-1-AP, proteintech), KRT13 (A7697, ABclonal), GAPDH (60004-1-lg, proteintech), HA-tag (3724S, Cell Signaling Technology), YBX1 (A7704, ABclonal), ALYREF (A6010, ABclonal), and β-actin (A3854, Sigma-Aldrich).

### Immunohistochemistry

The slides were put into the dewaxing solution I/II, 100% ethanol, 90% ethanol, 85% ethanol, and 75% ethanol for 5 min, respectively. Antigen retrieval was performed in Tris-EDTA (pH 9.0) using heat-induced protocol. The slides were incubated at 4°C with NSUN2 antibody (20854-1-AP, proteintech,1:400) overnight.

### Dot Blot

The total RNA of cells was extracted with RNAiso Plus (9109, Takara), followed by using the Magnetic mRNA Isolation Kit (s1550s, New England Biolabs) to purify polyadenylated mRNA twice according to the manufacturer. After 5-min denaturation at 95°C, the same amounts of serially diluted mRNA were loaded onto an Amersham Hybond N + membrane (RPN303B, GE Healthcare). After UV crosslinking twice for 2 min each time, membrane was stained with Methylene blue (A610622-0025, Sangon Biotech) according to the protocols of the manufacturer and was washed by 1× PBST. After the scanning to indicate the loading RNA, membrane was blocked with 5% non-fat milk in 1×PBST for 1 h at room temperature and then was incubated with anti‐m^5^C antibody (A19841, ABclonal) overnight (4°C). Membrane was washed by 1×PBST and was incubated by HRP-conjugated Affinipure goat anti-rabbit IgG (A0277, Beyotime) diluted 1:5,000 for 1 h at room temperature.

### Cell Viability Assays

*In vitro* cell viability was detected by Cell Counting Kit-8 (40203ES80, YEASEN) and real-time cell analysis (RTCA). For CCK-8 detection, 5×10^4^ cells were seeded into a 96-well plate per well and pre-incubated overnight at 37°C, 5% CO_2_. After that, 10 μl of CCK-8 solution was added to each well, and the absorbance at 450 nM was measured after 2-h incubation using SpectraMax^®^ Absorbance reader CMax plus. RTCA was performed by the real-time cell analyzer (xCELLigence RTCA SP, Roche) according to the operation manual. In brief, the same amounts of cells (about 5×10^4^) in 150 μl of medium containing 10% FBS were seeded in E-Plate 96. Relative cell counts were recorded and analyzed by xCELLigence RTCA SP for 48 h.

### Cell Migration and Invasion Assays

Cell migration and invasion capabilities were evaluated *via* transwell assays. The same amounts of cells were resuspended with serum-free Opti-MEM medium and then added to the upper chamber coated with or without Matrigel (BD Biosciences) after transfection. DMEM, RPMI1640, or MCCOY’S 5A with 20% FBS was added to the lower chambers as an attractant. After incubation for 8 h for migration or 24 h for invasion of Siha cells (24 h for migration or 48 h for invasion of CaSki cells and SKOV3 cells) at 37°C, non-migrating or non-invading cells were gently erased. The bottoms of the upper chambers were fixed and stained with 0.1% Crystal violet solution for 30 min. Then, three random fields were taken under a 100-fold microscope. Cell migration was also evaluated by wound healing assay. In brief, the same amounts of cells were seeded in 6-well plates. Three scratches were made using 200-μl pipette tips. After washing the plates three times with phosphate buffered saline (PBS), the cell was cultured in medium containing 2% FBS. The degrees of cell migration were recorded by microscopy at 0 and 24 h and analyzed by Image J.

### Cell Cycle Assays

Cell cycle was detected by using Cell cycle staining buffer (70-CCS01, MultiSciences) according to the protocols of the manufacturer. Briefly, the treated cells were harvested by trypsinization and washed with PBS, and then the cells were stained by 1 ml of DNA Staining solution and 10 μl of Permeabilization solution for 30 min. After staining, cell cycle analysis was performed by using CytoFLEX LX (Beckman Coulter Life Sciences).

### Statistics

All data in the manuscript represent three independent experiments giving similar results. Data are presented as mean ± standard deviation (SD). We performed the statistical analysis using GraphPad Prism 6 software. Kaplan–Meier curves were based on the log-rank test. The HR was performed using the Cox model. The significance between groups was determined using Student’s unpaired *t*-test. Statistical significance is reported as ***p < 0.001.

## Results

### *NSUN2* RNA Was Upregulated in Ovarian and Cervical Cancers

**Figure 1:**
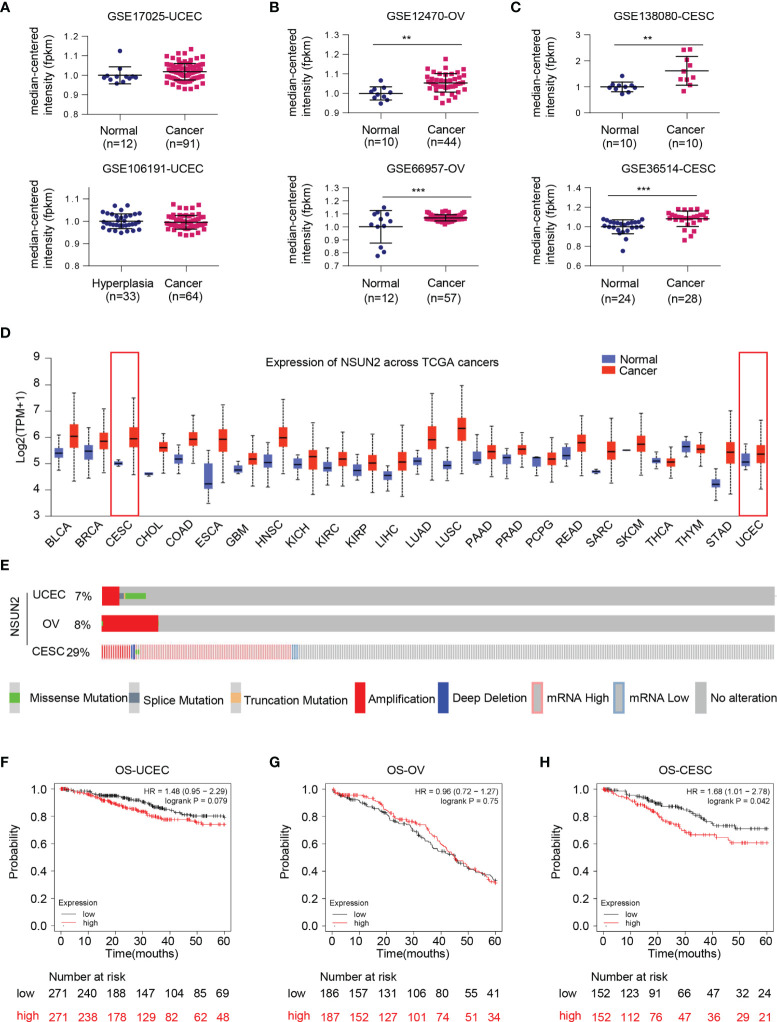
Bioinformatics analysis regarding expression status of NSUN2 gene in gynecological cancer. **(A)** Dot plot of the expression level of *NSUN2* mRNA in uterine corpus endometrial cancer (UCEC) tissues from GSE17025 and GSE106191. **(B)** Dot plot of the expression level of *NSUN2* in ovarian cancer (OV) tissues from GSE12470 and GSE66957. **(C)** Dot plot of the expression level of *NSUN2* in cervical squamous cell carcinoma (CESC) tissues from GSE138080 and GSE36514. **(D)** Pan-cancer expression status of *NSUN2* mRNA (normal vs. tumor) in TCGA database assessed by UALCAN website. **(E)** OncoPrint of c-BioPortal showing the proportions of each mutation type of NSUN2 in three gynecological cancers from TCGA samples. **(F–H)** Association of *NSUN2* RNA expression with 5-year overall survival (OS) in microarray data in all UCEC patients. **(F)** OV patients **(G)** and CESC patients **(H)** determined using KM-plotter online survival analysis tool. (Data shown are mean ± SEM, *n* = 3, Student’s unpaired *t*-test was used for statistical analysis, the log-rank test was used for Kaplan–Meier curves. The HR was performed using the Cox model. **p < 0.01; ***p < 0.001).

We primarily evaluated the role of NSUN2 in gynecological cancers through collecting the gene expression profile from multiple databases and websites including the GEO database, UALCAN website, oncomine, cBioPortal, and Kaplan–Meier Plotter. The results from GEO datasets showed no statistically significant difference of *NSUN2* expression in uterine corpus endometrial cancer (UCEC) (**Figure 1A**), while *NSUN2* is significantly upregulated in ovarian cancer (OV) and cervical squamous cell carcinoma (CESC) (**Figures 1B, C**). Similar results were found on *NSUN2* expression from TCGA database assessed by UALCAN website (**Figure 1D**). Interestingly, cBioPortal database showed lower proportion of NSUN2 alteration in UCEC (7%) and OV (8%) as compared with that of CESC (29%) (**Figure 1E**). Additionally, the Kaplan–Meier plot of 5-year overall survival curves stratified by *NSUN2* expression revealed a significant relationship between *NSUN2* expression and 5-year overall survival in CESC but not UCEC and OV (**Figures 1F**–**H**). Taken together, these findings suggest that NSUN2 likely contributes to the carcinogenesis of ovarian cancer and cervical cancer but not endometrial cancer. Thus, we conducted further study on ovarian cancer and cervical cancer.

### Role of NSUN2 in Ovarian Cancer

Consistent with upregulation of gene expression, the protein level of NSUN2 was also increased in ovarian cancer (**Supplementary Figure S1A**). In addition, NSUN2 protein level was positively associated to the individual cancer stages (**Supplementary Figure S1B**). Then, we examined the expression levels of NSUN2 in a set of clinical samples and found that *NSUN2* mRNA was highly expressed in ovarian cancer tissues (*n* = 20) compared to normal ovary tissues (*n* = 11) (**Supplementary Figure S2A**). Meanwhile, 4 out of 6 patient samples exhibited higher expression of NSUN2 protein in tumor tissues than their paired adjacent normal tissues as examined by Western blot (**Supplementary Figure S2B**). These results demonstrated that both transcript and protein levels of NSUN2 were upregulated in ovarian cancer.

To further explore the role of upregulated NSUN2 in ovarian cancer, we established two NSUN2-knockdown stable clones in ovarian cancer cell lines A2780 and SKOV3 (**Supplementary Figures S2C, D**). NSUN2 knockdown has marginal effects on cell proliferation (**Supplementary Figures S2E, F**) and cell cycle distribution (**Supplementary Figures S2G, H**). The wound-healing assay also showed no obvious change of migration in the NSUN2 knockdown clones (**Supplementary Figure S2I**). Taken together, these results suggest that the overexpression of NSUN2 in ovarian cancer may be an incidental event.

### NSUN2 Upregulation Promotes Migration and Invasion of Cervical Cancer Cells in an m^5^C-Dependent Manner

**Figure 2:**
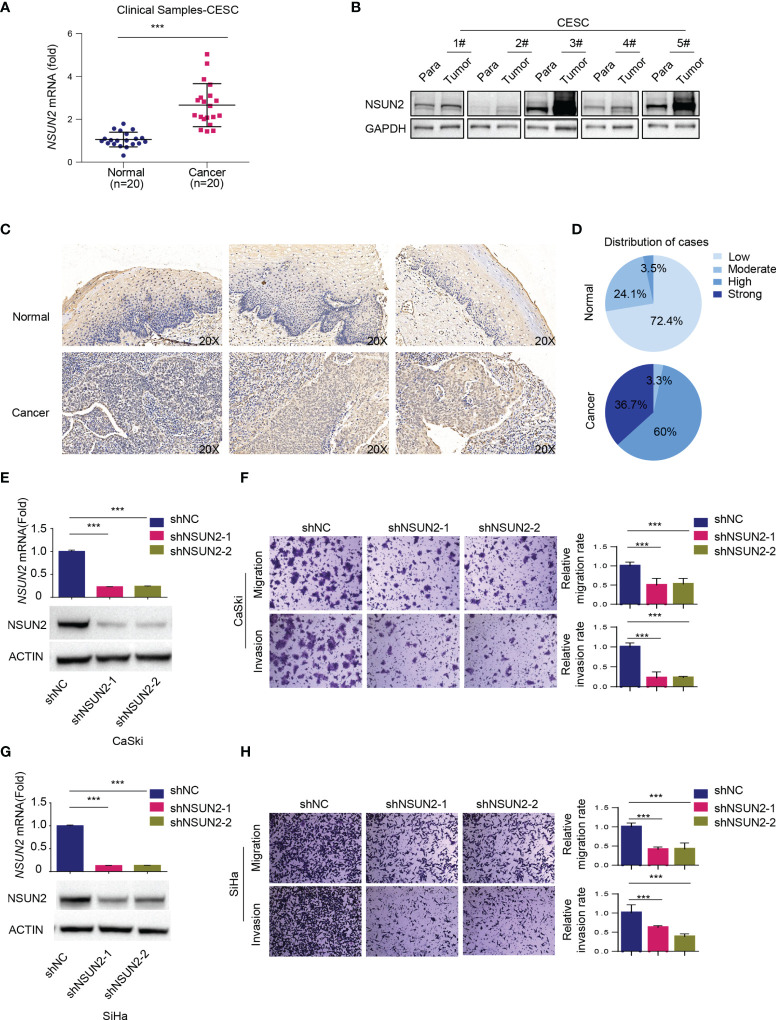
Role of NSUN2 in cervical cancer. **(A)** The expression of *NSUN2* mRNA in 20 cervical cancer tissues and 20 normal cervical tissues from clinical samples as assessed by RT-qPCR. **(B)** The expression of NSUN2 protein in 5 clinical samples with paired adjacent normal tissues in cervical cancer by Western blot. **(C, D)** Representative images **(C)** and quantification **(D)** of NSUN2 immunohistochemistry from tissue chip consisting of 29 normal cervix and 30 cervical cancer paraffin sections. **(E, G)** Validation of NSUN2 knockdown efficacy of in CaSki **(E)** and SiHa cells **(G)** by RT-qPCR and Western blot. **(F, H)** The effects of NSUN2 knockdown on migration and invasion as measured by transwell assays in CaSki **(F)** and SiHa **(H)** cells. Representative images (left panel) and quantification (right panel) of transwell assays showed the migration and invasion capability of CaSki and SiHa cells. (Data shown are mean ± SEM, *n* = 3; Student’s unpaired *t*-test was used for statistical analysis, ***p < 0.001).

As mentioned above, bioinformatics analysis showed that NSUN2 was upregulated and negatively related to overall survival in cervical cancer (**Figures 1C, D, H**). In line with this, we found that *NSUN2* mRNA level was significantly upregulated in cervical cancer tissues (*n* = 20) compared to normal cervix samples (*n* = 20) (**Figure 2A**), and NSUN2 protein levels were also higher in randomly selected tumor tissues (*n* = 5) than their paired adjacent normal tissues (**Figure 2B**). We next collected 29 normal cervix and 30 cervical cancer paraffin sections to prepare tissue chip, and the immunohistochemistry analysis showed higher levels of NSUN2 in cervical cancer than in normal tissues (**Figures 2C, D** and **Supplementary Figure S3A**). These results indicate that both mRNA and protein levels of NSUN2 were upregulated in cervical cancer.

To investigate the biological function of highly expressed NSUN2 in cervical cancer, we then established NSUN2 knockdown stable clones in two common cervical cancer cell lines CaSki and SiHa (**Figures 2E, G**). CCK-8 and real-time cell analysis (RTCA) assays showed that knockdown of NSUN2 has little effect on cervical cancer cell proliferation (**Supplementary Figures S3B**–**E**), and downregulation of NSUN2 led to minor changes in cell cycle distribution (**Supplementary Figures S3F, G**). In contrast, depletion of NSUN2 significantly inhibited migration and invasion of CaSki and SiHa cells in transwell assays (**Figures 2F, H**). These results suggest that NSUN2 plays an important role in migration and invasion but not proliferation in cervical cancer.

**Figure 3:**
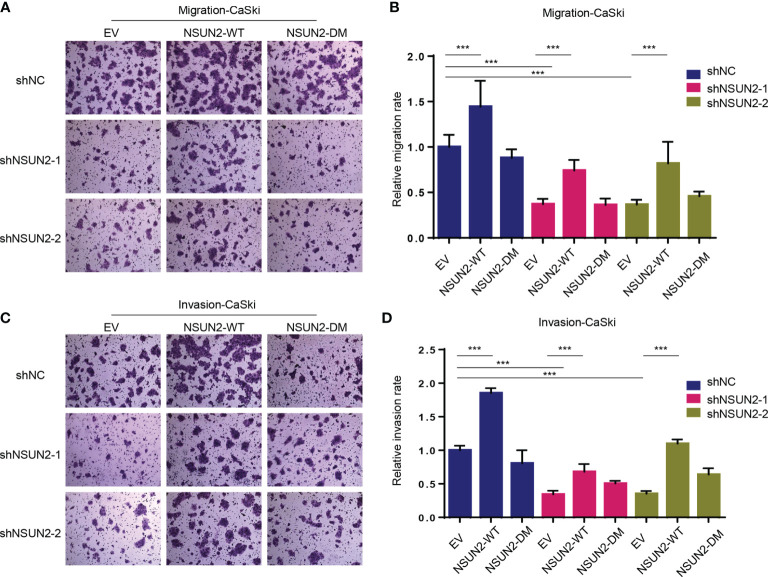
NSUN2 promotes migration and invasion of cervical cancer cells in an m^5^C-dependent manner. **(A, B)** Effect of wild-type or mutant NSUN2 on cell migration in NSUN2 knockdown CaSki cells. Representative images **(A)** and quantification **(B)** of transwell assays showed the migration capability of CaSki cells. **(C, D)** Effect of wild-type or mutant NSUN2 on cell invasion in NSUN2 knockdown CaSki cells. Representative images **(C)** and quantification **(D)** of transwell assays showed the invasion capability of CaSki cells. (EV, empty vector; data shown are mean ± SEM, *n* = 3, Student’s unpaired *t*-test was used for statistical analysis, ***p < 0.001).

Downregulation of NSUN2 significantly decreased m^5^C levels in CaSki and SiHa cells (**Supplementary Figures S4A, B**). We next asked whether the effect of NSUN2 on migration and invasion is m^5^C dependent. For this end, we constructed wild-type NSUN2 (NSUN2-WT) expression vector and catalytically inactive mutant NSUN2 (NSUN2-DM) expression vector harboring double point mutations in its releasing (cysteine 271) and catalytic (cysteine 321) sites. Knockdown of NSUN2 in CaSki cells led to the inhibition of cell migration and invasion, and compensation of NSUN2-WT but not NSUN2-DM could reverse the inhibition of cell migration and invasion (**Figures 3A**–**D**), suggesting that promotion of cell migration and invasion by upregulated NSUN2 is dependent on its m^5^C RNA methyltransferase activity. In other words, NSUN2 promoted cervical cancer migration and invasion in an m^5^C-dependent manner.

### KRT13 Plays an Important Role in NSUN2-Mediated Cell Migration and Invasion in Cervical Cancer Cells

**Figure 4:**
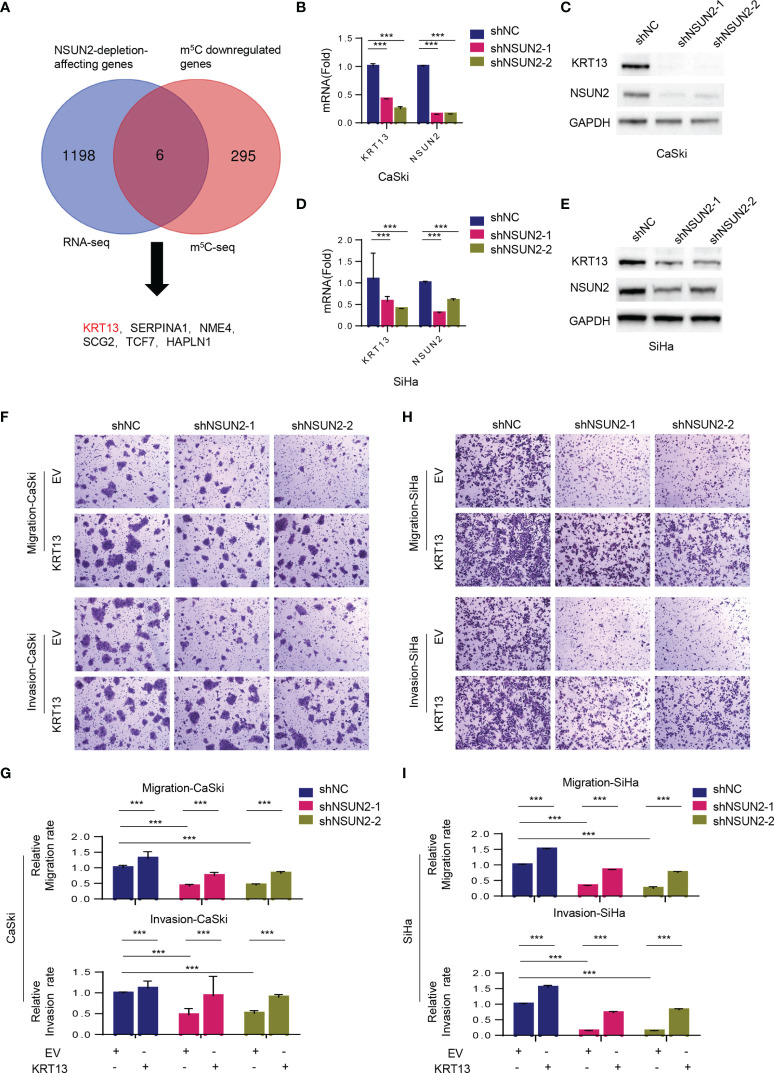
NSUN2 promotes cell migration and invasion in cervical cancer *via* KRT13. **(A)** Venn diagram showing 6 overlapping genes between RNA-BisSeq and RNA-Seq data. **(B–E)** RT-qPCR and Western blot showing *KRT13* mRNA and protein level in NSUN2-knockdown CaSki **(B, C)** and SiHa cells **(D, E)**. **(F, G)** Effect of KRT13 overexpression on cell migration (upper panel) and invasion (lower panel) in NSUN2 knockdown CaSki cells. Representative images **(F)** and quantification **(G)** of transwell assays showed the migration and invasion capability of CaSki. **(H, I)** Effect of KRT13 overexpression on cell migration (upper panel) and invasion (lower panel) in NSUN2 knockdown SiHa cells. Representative images **(H)** and quantification **(I)** of transwell assays showed the migration and invasion capability of SiHa. (EV, empty vector; data shown are mean ± SEM, *n* = 3, Student’s unpaired *t*-test for statistical analysis, ***p < 0.001).

We then performed RNA-BisSeq and RNA-Seq in wild-type and NSUN2 depleted CaSki cells to explore the potential m^5^C-dependent pathways involved in NSUN2-mediated cell migration and invasion. The RNA-BisSeq results showed that knockdown of NSUN2 significantly reduces the number of m^5^C sites of the transcripts across chromosomes, especially across chromosomes 1, 11, 12, and 19 (**Supplementary Figure S4C**). After sequence context comparison, we found that NSUN2 has no preferred motif among CHH, CHG, and CG (H=A, C, U) in CaSki cells, because there was no significant alteration of sequence distribution proportion between control (shNC) and NSUN2 knockdown cells (**Supplementary Figures S4D, E**). On the other hand, through profiling analysis, we found downregulation of m^5^C levels on 301 genes, and further identified six genes overlapping between 1204 NSUN2-depletion-affecting genes and 301 m^5^C downregulated genes (**Figure 4A**). Next, we verified expression of these genes in the other cervical cancer cell line, SiHa, and found that only *KRT13* levels displayed significant downregulation in NSUN2-depleted SiHa cells as well (**Supplementary Figure S4F**). Furthermore, besides mRNA levels, the expression of KRT13 protein was also obviously downregulated in NSUN2 knockdown CaSki and SiHa cells (**Figures 4B**–**E**), suggesting *KRT13* mRNA as the downstream target gene of NSUN2-m^5^C regulatory pathway in cervical cancer cells. Supporting this notion, inhibition of migration and invasion by NSUN2 knockdown was significantly reversed by overexpression of KRT13 in both CaSki and SiHa cells (**Figures 4F**–**I** and **Supplementary Figures S4G, H**). However, overexpressing NSUN2 cannot rescue cell migration in KRT13 knocking down CaSki cells (**Supplementary Figures S5A, B**), which indicated that NSUN2 regulates cell migration in a KRT13-dependent manner. Collectively, our results demonstrated that NSUN2 promotes cervical cancer cell migration and invasion *via* a KRT13-dependent manner.

### Identification of a Novel NSUN2-Dependent m^5^C-YBX1-KRT13 Oncogenic Regulatory Pathway

**Figure 5:**
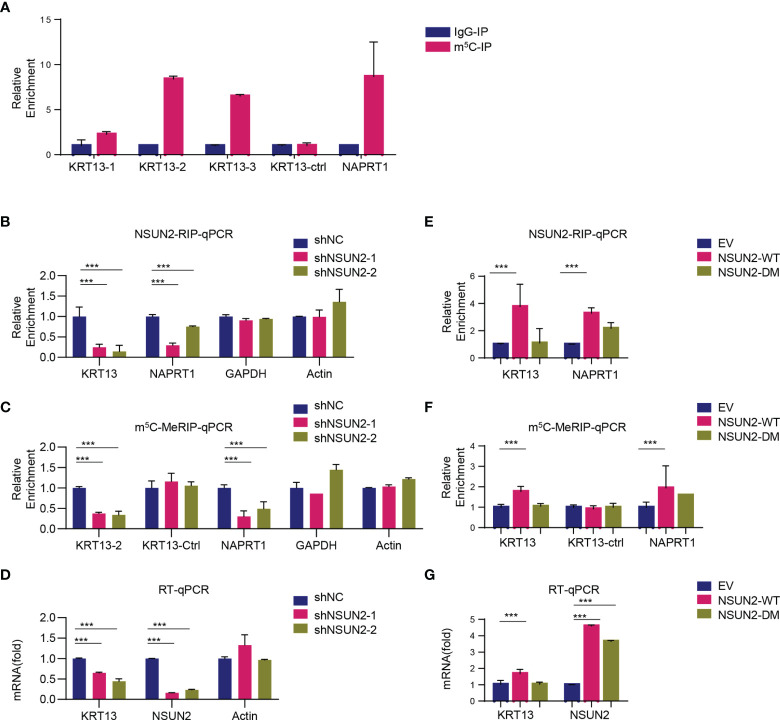
*KRT13* mRNA is enriched of m^5^C modification. **(A)** m^5^C modification on *KRT13* mRNA analyzed by m^5^C-MeRIP-qPCR in CaSki cells. *NAPRT1* was used as a positive control. **(B–D)** RIP analysis of NSUN2-*KRT13* interaction **(B)** and m^5^C-MeRIP-qPCR detection of m^5^C level on *KRT13* **(C)** along with RT-qPCR analysis of *KRT13* mRNA level **(D)** in NSUN2 knockdown and control CaSki cells. GAPDH and Actin were used as a negative control. **(E–G)** RIP analysis of NSUN2-*KRT13* interaction **(E)** and m^5^C-MeRIP-qPCR detection of m^5^C level on *KRT13* **(F)** along with RT-qPCR analysis of *KRT13* mRNA level **(G)** in wild-type and mutant NSUN2 overexpressed CaSki cells. (Data shown are mean ± SEM, *n* = 3; Student’s unpaired *t*-test was used for statistical analysis, ***p < 0.001).

We next investigated whether and how NSUN2 regulates *KRT13* transcripts *via* m^5^C modification. The RNA-BisSeq results showed that there were multiple m^5^C modification sites on 3’-UTR and CDS regions of *KRT13* mRNA (**Supplementary Figure 6A**). Among these, three clusters (marked green in **Figure 5A**) were demethylation in NSUN2 knockdown CaSki cells. We designed 3 pairs of primers specifically amplifying these m^5^C clusters on *KRT13* mRNA (named KRT13-1, KRT13-2, and KRT13-3) and a pair of primers amplifying the non-m^5^C region (named KTR13-ctrl) (**Supplementary Figure 6A**). m^5^C-MeRIP-qPCR results showed high enrichment of m^5^C modification on *KRT13* mRNA in CaSki cells, especially at the KRT13-2 (CDS region near 3’-UTR) and KRT13-3 (3’-UTR region); *NAPRT1*, a gene validated to harbor m^5^C modification (7), was used as a positive control (**Figure 5A**). Knockdown of NSUN2 significantly reduced the interaction between NSUN2 and *KRT13* mRNA (**Figure 5B**) accompanied by a notable reduction of m^5^C levels of *KRT13* (**Figure 5C**), leading to an obvious downregulation of *KRT13* mRNA levels (**Figure 5D**). GAPDH and Actin were used as negative control. Furthermore, overexpression of wild-type NSUN2 (NSUN2-WT) increased the interaction between NSUN2 and *KRT13* mRNA (**Figure 5E**), accompanied by a notable upregulation of *KRT13* m^5^C modification (**Figure 5F**), leading to a significant increase of *KRT13* mRNA level (**Figure 5G**). Remarkably, overexpression of catalytically inactive mutant NSUN2 (NSUN2-DM) neither stimulated m^5^C levels of *KRT13* nor increased *KRT13* mRNA expression (**Figures 5F, G**). These results suggest that NSUN2 binds with and catalyzes m^5^C modification of *KRT13* to upregulate *KRT13* mRNA.

### NSUN2 Stabilizes *KRT13* mRNA by Recruiting YBX1

**Figure 6:**
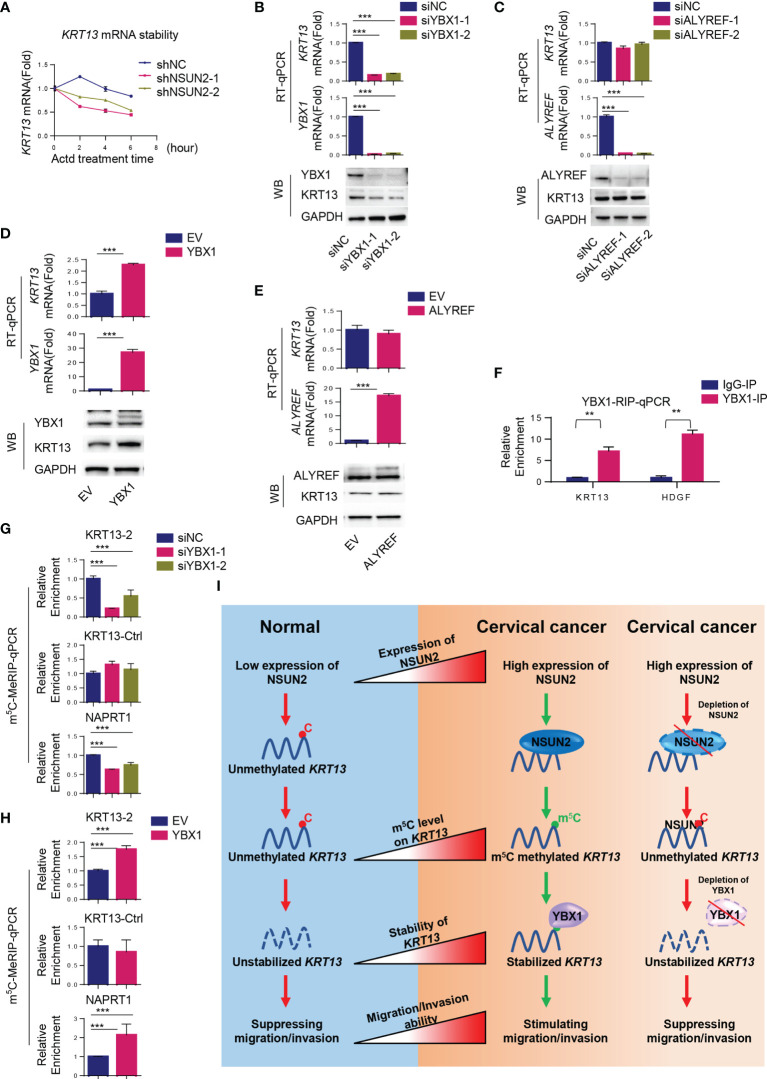
NSUN2 stabilizes *KRT13* mRNA by recruiting YBX1. **(A)** Effect of NSUN2 on the stability of *KRT13* mRNA. CaSki cells transfected with two NSUN2-targeting shRNAs were treated with 10 μg/ml RNA synthesis inhibitor Actinomycin D (Actd) for indicated time. Level of *KRT13* mRNA was analyzed by RT-qPCR. **(B, C)** Effect of YBX1 **(B)** and ALYREF **(C)** knockdown on *KRT13* mRNA and protein level in CaSki cells as assessed by RT-qPCR and Western blot. **(D, E)** Effect of YBX1 **(D)** and ALYREF **(E)** overexpression on *KRT13* mRNA and protein level in CaSki cells as assessed by RT-qPCR and Western blot. **(F)** RIP-qPCR analysis of YBX1-*KRT13* interaction. IgG was used as a negative control. **(G, H)** Effect of YBX1 on m^5^C modification occurred on *KRT13* mRNA. YBX1 was silenced **(G)** or overexpressed **(H)** in CaSki cells, and the m^5^C modification on *KRT13* mRNA was measured by m^5^C-MeRIP-qPCR. *NAPRT1* was used as a positive control. *KRT13*-Ctrl was used as a negative control. **(I)** A working model of the mechanism by which NSUN2 promotes cervical cancer migration and invasion. (Data shown are mean ± SEM, *n* = 3; Student’s unpaired *t*-test was used for statistical analysis, **p < 0.01, ***p < 0.001).

We next investigated the mechanism by which NSUN2 upregulated *KRT13* mRNA. The mRNA stability assay showed that depletion of NSUN2 resulted in destabilization of *KRT13* mRNA, suggesting that m^5^C regulates the stability of *KRT13* mRNA (**Figure 6A**). YBX1 and ALYREF are two well-characterized m^5^C readers that could regulate m^5^C mRNA stability and transport, respectively (26). We found that siRNA knockdown of YBX1 but not ALYREF decreases *KRT13* mRNA and protein levels (**Figures 6B, C**), suggesting that YBX1 is the reader protein involved in NSUN2 mediated stabilization of *KRT13*. Supporting this notion, overexpression of YBX1 but not ALYREF enhanced the levels of *KRT13* mRNA and its protein (**Figures 6D, E**); RNA stability assay further demonstrated that suppression of YBX1 indeed reduced the stability of *KRT13* mRNA (**Supplementary Figures S6B, C**). Inhibiting RNA synthesis by using Actinomycin D (Actd), a transcription inhibitor, cannot abolish the upregulation of *KRT13* mRNA induced by YBX1 overexpression (**Supplementary Figure S6D**). Meanwhile, the KRT13 promoter reporter assay showed YBX1 knocking down cannot decrease the activity of KRT13 promoter (**Supplementary Figure S6E**). Collectively, these results demonstrated that rather than regulating *KRT13 t*ranscription, YBX1 modulated KRT13 expression by regulating its RNA stability. Moreover, YBX1-RIP assay confirmed the binding of YBX1 to *KRT13* mRNA (**Figure 6F**), and knocking down NSUN2 can significantly decrease the binding between YBX1 and *KRT13* mRNA (**Supplementary Figure S6F**), further demonstrating that YBX1 regulates KRT13 in a NSUN2-dependent manner. HDGF was used as a positive control (9). Furthermore, m^5^C-MeRIP-qPCR results showed that knockdown of YBX1 decreases while overexpression of YBX1 increases m^5^C levels on *KRT13* mRNA (**Figures 6G, H**). We performed a rescue experiment and found that overexpressing NSUN2 could not rescue the expression levels of *KRT13* mRNA in YBX1 depleted CaSki cells (**Supplementary Figure S6G**), which indicated that NSUN2 regulates *KRT13* mRNA in a YBX1-dependent manner. Together, these results suggest that NSUN2 promotes m^5^C modification on *KRT13*, and m^5^C methylated *KRT13* would be recognized and stabilized by the m^5^C reader protein YBX1. **Figure 6I** shows the working model by which NSUN2 stabilizes *KRT13 via* YBX1, through which it regulates cervical cancer invasion and migration.

## Discussion

Cervical, ovarian, and endometrial cancers are three major gynecological cancers with high tissue homology. Currently, surgical dissection and intensive chemotherapy remain the main treatment approach for patients carrying these cancers and might severely affect fertility and quality of life (27). Thus, novel molecular markers and targeted treatment options are urgently needed. m^5^C modification ubiquitously occurs on mammalian mRNAs and plays important roles in multiple biological processes (7, 13, 28). Currently, the role of m^5^C in cancer initiation and progression is gaining more and more research attention, but the detailed mechanism remains unclear (13). Although NSUN2 is the main methyltransferase for m^5^C methylation on mRNA (8, 29), we extended to explore the expression of all potential m^5^C methyltransferases (NOP2, NSUN3, 4, 5, 6, 7, and DNMT2) in cervical cancer, ovarian cancer, and endometrial cancer (**Supplementary Figure S7**). Among these results, we only found a significant reduction of *NSUN6* mRNA in ovarian cancer (**Supplementary Figure S7A**). However, no difference of the expression of NSUN6 protein was found between ovarian cancer and normal tissue by the CPTAC database (http://ualcan.path.uab.edu/cgi-bin/CPTAC-Result.pl?genenam=NSUN6&ctype=OV) (**Supplementary Figure S7B**). In addition, the expression of NSUN6 was not correlated with the prognosis of ovarian cancer analyzed by Kaplan–Meier Plotter (**Supplementary Figures S7C, D**). As an m^5^C methyltransferase with unique mRNA catalytic activity, NSUN2 was found to be highly expressed in multiple cancers including gastric, bladder, gallbladder, and breast cancers (9, 13, 14, 30–33), suggesting that NSUN2 might exert oncogenic properties through affecting m^5^C levels in cancer cells. Therefore, we investigated whether and how NSUN2/m^5^C played a role in the major gynecological (cervical, ovarian, and endometrial) cancers. Although NSUN2 expression was not changed in endometrial carcinoma, and the upregulation of NSUN2 in ovarian cancer may be an incidental event as cell proliferation, cell cycle distribution, and cell migration were not affected upon NSUN2 depletion, we demonstrated a novel NSUN2-dependent m^5^C-*KRT13*-YBX1 oncogenic regulatory pathway in cervical cancer, displaying the diversity and complexity of gynecological cancers.

These findings echoed a previous study demonstrating by pan-cancer analysis that although there were many similarities among these three major gynecologic cancers, substantial differences exist (34). For example, it is reported that most of the uterine corpus endometrial cancer (UCEC) samples show little immune infiltration while most of the cervical squamous cell carcinoma (CESC) samples show high immune marker signatures; the lncRNA profiles are also very distinct among UCEC, CESC, and OV (34). Moreover, the risk factors of these major gynecologic cancers are also quite different. To be specific, the endometrial cancer and ovarian cancer have been considered to be related to hormone imbalance and obesity (20, 22, 35), while the main risk factors for cervical cancer is the persistent infection of HPV (18, 36). These cellular context and epigenetic differences may lead to the varying roles of NSUN2 in these cancers. Interestingly, the results of GO analysis showed that alteration of NSUN2 would affect m^5^C methylation on genes involved in viral process pathways (**Supplementary Figure S8A**), which suggest the interplay between NSUN2-mediated m^5^C modification and HPV infection, and would be a reason, in part, why NSUN2 plays a unique role to stimulate malignancy in cervical cancer but not in ovarian and endometrial cancers.

We identified keratin 13 (*KRT13*) mRNA as the downstream target gene of NSUN2 in cervical cancer, which contributes to the activity of tumor cell migration and invasion. Keratin is a family of intermediate filament proteins expressed in a cell-specific and differentiation-dependent manner and was encoded by more than 50 genes on chromosomes 12 and 17 (37). KRT13 is a 54-kDa type 1 acidic intermediate filament protein, which plays different roles in distinct cancers depending on the context. Some studies found that KRT13 serves as a tumor suppressor with low expression in oral dysplasia, esophageal squamous cell carcinoma, bladder cancer, and so on (38–40). In contrast, other studies also found that higher KRT13 levels in colorectal cancer, gastric cancer, and tongue squamous cell carcinoma may promote the development of these cancers (41), and KRT13 showed the activity to promote metastasis of breast cancer by altering the nuclear translocation of plakoglobin and modulating downstream c-Myc-dependent signaling (42). Although the potential oncogenic function of KRT13 has been reported, the mechanism by which *KRT13* is regulated in cancer cells is elusive. In this study, we not only demonstrated the ability of KRT13 to stimulate cervical cancer cell migration and invasion, but also revealed a novel upstream NSUN2-m^5^C-YBX1 pathway for regulation of *KRT13* mRNA stability and expression. These results suggest that NSUN2, YBX1, and KRT13 could serve as potential diagnostic markers or potential therapeutic targets in cervical cancer. On the other hand, through the GO analysis, we found that NSUN2 depletion-induced m^5^C downregulated genes were mainly involved in cell migration and invasion such as focal adhesion, cell substrate junction, cell adhesion molecule binding, cadherin binding, and regulation of actin cytoskeleton (**Supplementary Figures S8B**–**D**), consistent with our results that NSUN2 promotes cervical cancer cell migration and invasion (**Figure 2**). Since multiple pathways participate in regulation of migration and invasion, it is likely that NSUN2 could modulate migration and invasion *via KRT13* mRNA-independent mechanisms. Further investigation is needed for comprehensive understanding of the NSUN2-mediated regulation of cell migration and invasion.

The massive diversity of tumor gene expression and mutations is a well-known feature of cancer and one of the major challenges in development of cancer treatment approaches. However, the mechanisms remain largely unknown. In this study, we reported the distinct roles of NSUN2 in three major gynecological cancers and demonstrated an NSUN2-mediated m^5^C-dependent oncogenic pathway, which improved the understanding of tumor diversity and suggested that besides epigenetic modifications on histone and DNA, m^5^C-modified RNA contributes to tumor diversity as well.

## Data Availability Statement

The datasets presented in this study can be found in online repositories. The names of the repository/repositories and accession number(s) can be found in the article/**Supplementary Material**.

## Ethics Statement

The studies involving human participants were reviewed and approved by the Institutional Review Board (or Ethics Committee) of Women’s Hospital, School of Medicine, Zhejiang University. The patients/participants provided their written informed consent to participate in this study.

## Author Contributions

Conceptualization: C-HH and XC. Methodology: C-HH and XC. Software: SL and YS. Validation: LW, JZ, and YS. Formal analysis: JZ. Investigation: LW, JZ, YS, and ZS. Resources: SY, SS, and FW. Data curation: YM and GZ. Writing—original draft preparation: LW, JZ, and YS. Writing—review and editing: YM. Visualization: LW. Supervision: C-HH and XC. Project administration: C-HH and XC. Funding acquisition: FW and XC. All authors contributed to the article and approved the submitted version.

## Funding

This research was funded by grants from Key Research and Development Program of Zhejiang province, Grant No. 2019C03010; the National Natural Science Foundation of China, Grant Nos. 31972883 and 8200015; the National Key Research and Development Program of China 2021YFC2700903; and the Zhejiang Provincial Natural Science Foundation, Grant No.LY21H160032.

## Conflict of Interest

The authors declare that the research was conducted in the absence of any commercial or financial relationships that could be construed as a potential conflict of interest.

The reviewer [QG] declared a shared affiliation with one of the authors [SL] to the handling editor at time of review.

## Publisher’s Note

All claims expressed in this article are solely those of the authors and do not necessarily represent those of their affiliated organizations, or those of the publisher, the editors and the reviewers. Any product that may be evaluated in this article, or claim that may be made by its manufacturer, is not guaranteed or endorsed by the publisher.
