# Supplementary material for: Distinct Roles of m5C RNA Methyltransferase NSUN2 in Major Gynecologic Cancers
Source: Front Oncol. 2022 Feb 25;12:786266. doi: 10.3389/fonc.2022.786266 (PMC8916577; doi:10.3389/fonc.2022.786266)
Supplement: Supplementary file 1 [file DataSheet_1.pdf]

Supplementary Material

Supplementary Table S1. Primer sequences.

Gene	Primer sequences（5’-3’）	
	**Forward sequence**	**Reverse sequence**
**NSUN2**	CAGTGGAGACGGCACTATGA	GACACATCAGCAAGCTCCAA
**YBX1**	GAGAAGTGATGGAGGGTGCT	TTAGGGTTTTCTGGGCGTCT
**ALYREF**	AAGGCCATGAAGCAGTACAACG	GAATTCCTGCCGGCACCTCT
**SERPINA1**	GACAGAAGGTCTGCCAGCTT	CTTGGAGAGCTTCAGGGGTG
**HAPLN1**	CCAGGGTAGAGTGTTTCTGAAGG	CCCATAATCTTCCAGAGTGAGGT
**NME4**	AGGGTACAATGTCGTCCGC	GACGCTGAAGTCACCCCTTAT
**TCF7**	TTGATGCTAGGTTCTGGTGTACC	CCTTGGACTCTGCTTGTGTC
**SCG2**	ACCAGACCTCAGGTTGGAAAA	AAGTGGCTTTCATCGCCATTT
**KRT13**	CCCCAGGCATTGACCTGAC	GTGTTGGTAGACACCTCCTTG
**GAPDH**	GCACCGTCAAGGCTGAGAAC	TGGTGAAGACGCCAGTGGA
**KRT13-1**	CCTCCTCACTGGCAATGAGA	GTCCCGGAGCTCTTCAATG
**KRT13-2**	AACACGGTGGCAGAGACG	GCGGTAGGTGGCGATCTC
**KRT13-3**	TTCCCTGCAGGAGAGAGGAG	AACCAAAGCGTATCCAGGTC
**KRT13-ctrl**	GTTTGTGTCCGGGGGATCAG	CGCCATCACAAGCACCAAAG
**NAPRT1**	GTGAGGTGAATGTCATTGGCA	CATGAGTGGAGACCCGTCAGA
**Luciferase**	ATGGAAGACGCCAAAAACAT	CGAACGGACATTTCGAAGTA

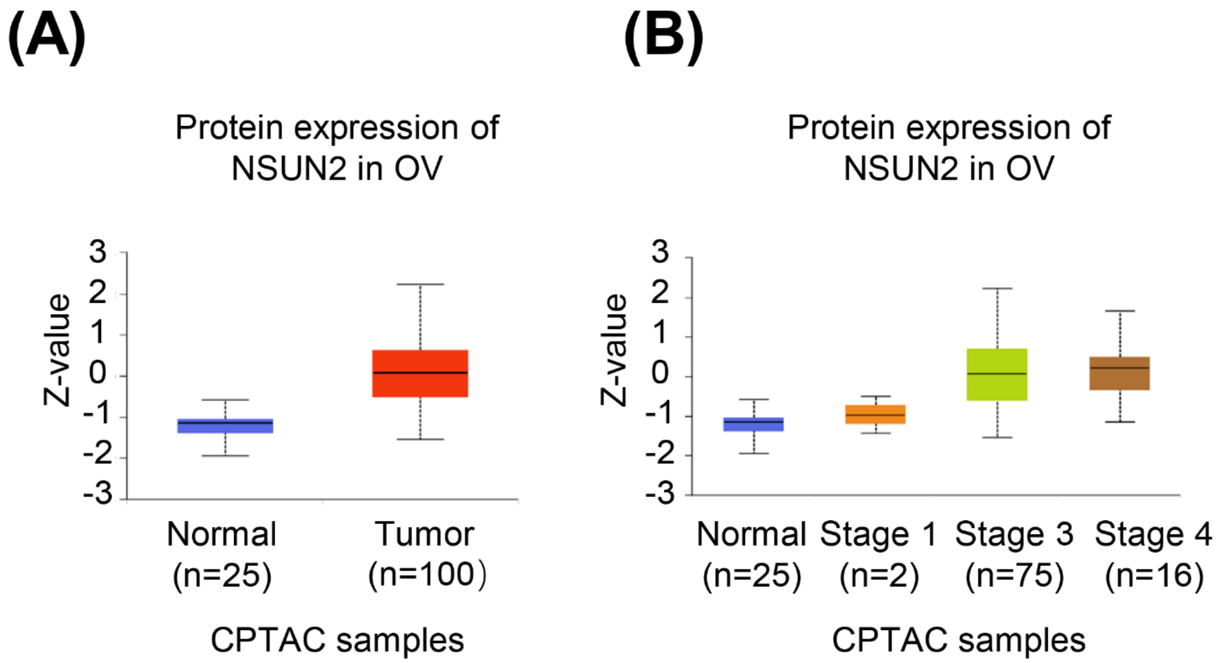

Supplementary Figure S1: **(A)** Box plot showing NSUN2 protein expression in ovarian tissues (normal vs. tumor) in CPTAC database assessed by UALCAN. **(B)** Box plot showing association between NSUN2 protein expression with individual cancer stages in ovarian cancer assessed by UALCAN website.

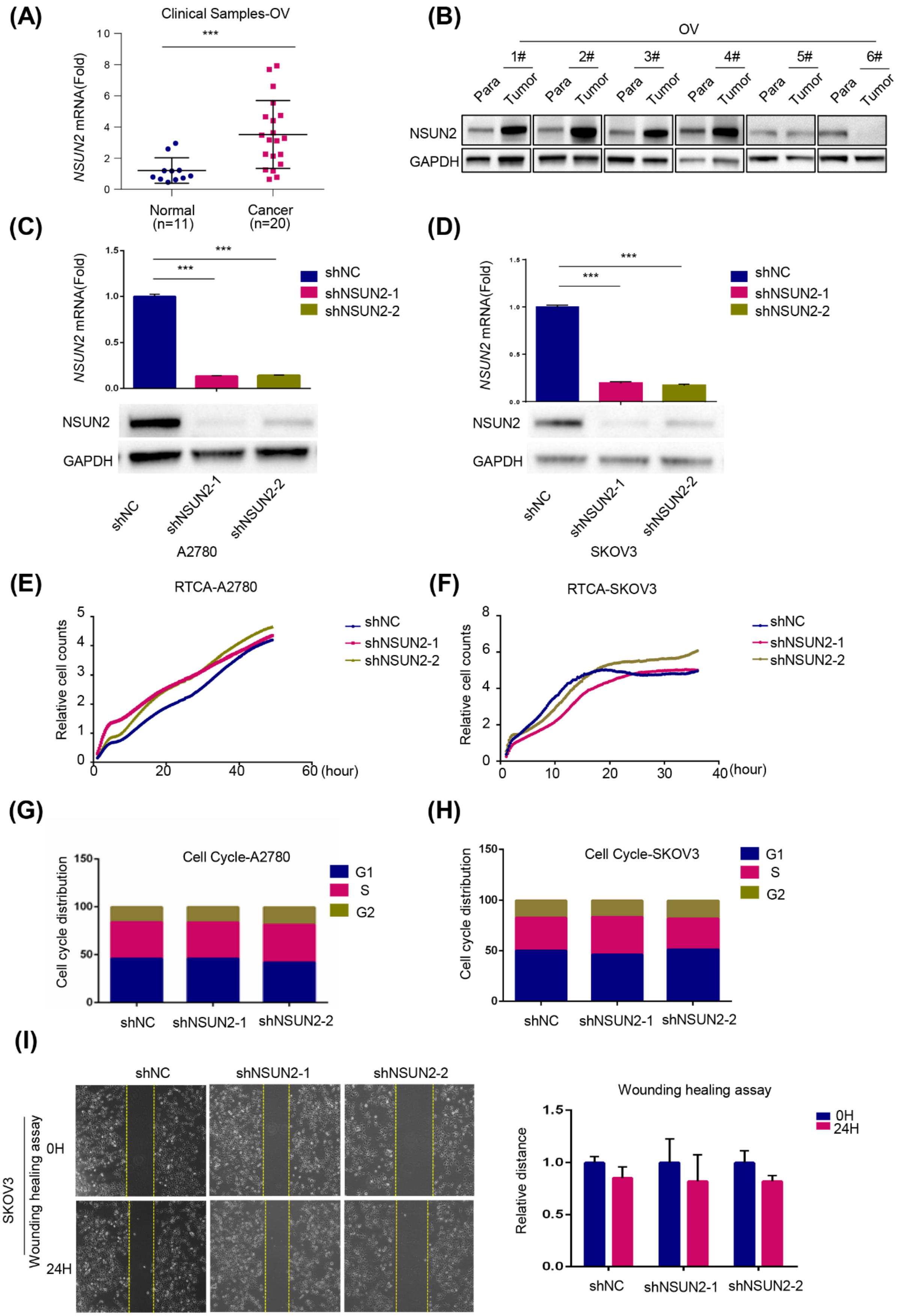
(A)
Clinical Samples-OV
***
NSUN2 mRNA(Fold)
Normal (n=11)
Cancer (n=20)
(B)
OV
1# 2# 3# 4# 5# 6#
Para Tumor
NSUN2
GAPDH
(C)
NSUN2 mRNA(Fold)
***
shNC
shNSUN2-1
shNSUN2-2
NSUN2
GAPDH
A2780
(D)
NSUN2 mRNA(Fold)
***
shNC
shNSUN2-1
shNSUN2-2
NSUN2
GAPDH
SKOV3
(E)
RTCA-A2780
Relative cell counts
(hour)
shNC
shNSUN2-1
shNSUN2-2
(F)
RTCA-SKOV3
Relative cell counts
(hour)
shNC
shNSUN2-1
shNSUN2-2
(G)
Cell Cycle-A2780
Cell cycle distribution
G1
S
G2
shNC shNSUN2-1 shNSUN2-2
(H)
Cell Cycle-SKOV3
Cell cycle distribution
G1
S
G2
shNC shNSUN2-1 shNSUN2-2
(I)
shNC shNSUN2-1 shNSUN2-2
SKOV3
Wounding healing assay
0H
24H
Wounding healing assay
Relative distance
0H
24H
shNC shNSUN2-1 shNSUN2-2

Supplementary Figure S2: **(A)** Dot plot of the expression of *NSUN2* mRNA in 20 ovarian cancer tissues and 11 normal ovarian tissues from clinical samples assessed by RT-qPCR. **(B)** The expression of NSUN2 protein in 6 clinical tumor samples with paired adjacent normal tissues in ovarian cancer by western blot. **(C, D)** Validation of the knockdown efficacy of NSUN2 in A2780 **(C)** and SKOV3 cells **(D)** by RT-qPCR (upper panel) and western blot (lower panel). **(E, F)** Effect of NSUN2 knockdown on cell proliferation in A2780 **(E)** and SKOV3 cell lines **(F)** as analyzed by RTCA assay. **(G, H)** Effect of NSUN2 stable knock down on cell cycle distribution in A2780 **(G)** and SKOV3 cell lines **(H)** analyzed by flow cytometry analysis. **(I)** Effect of NSUN2 knockdown on cell migration in SKOV3 cell lines as analyzed by wounding healing assay. Representative images (left panel) and quantification (right panel) of wounding healing assay showed the migration capability of SKOV3 cells. (RTCA: real-time cell analysis; data shown is mean ± SEM, n = 3; student's unpaired t-test was used for statistical analysis, *** $P < 0.001$.)

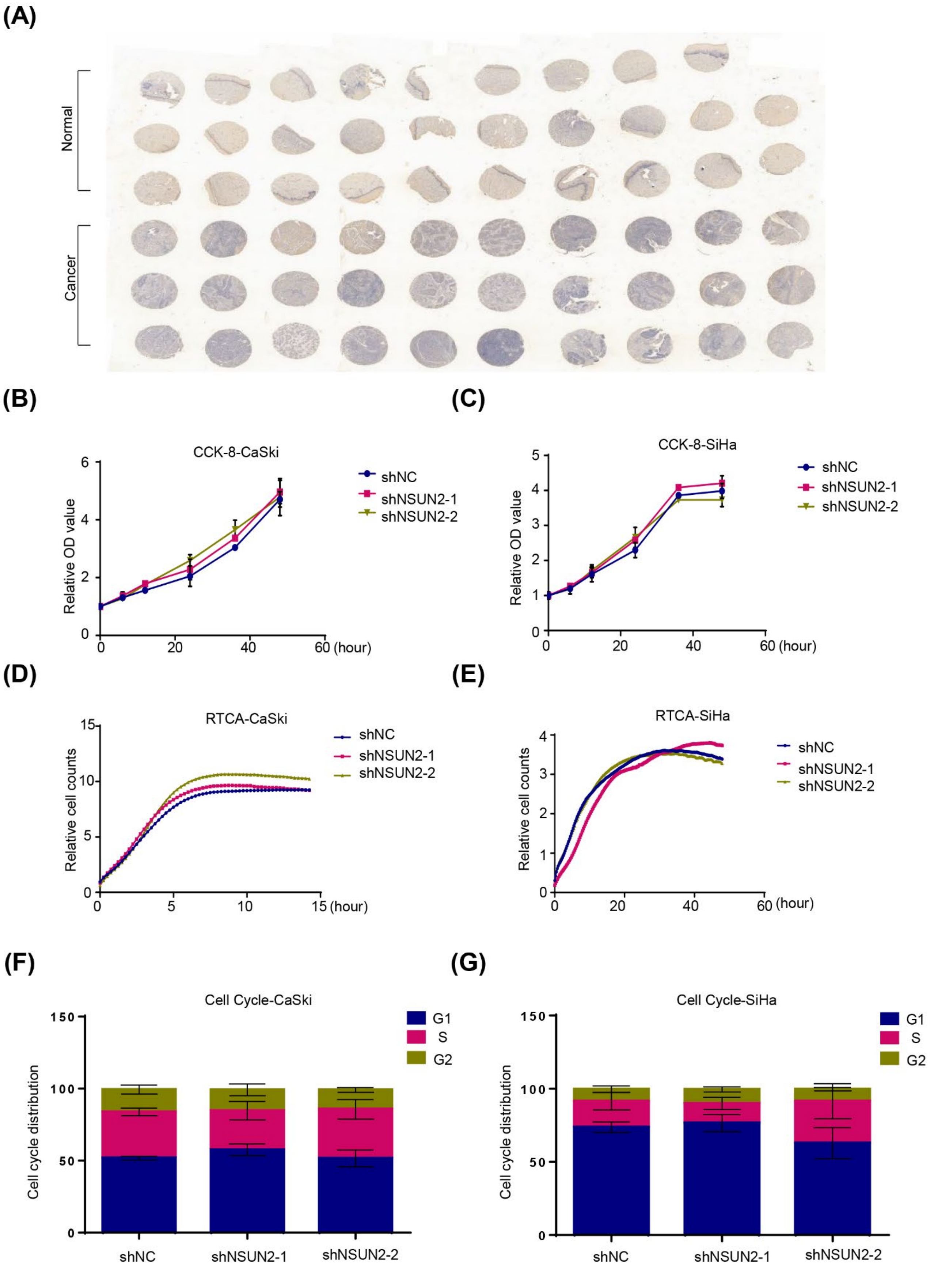
(A)
Normal
Cancer
(B)
(C)
CCK-8-CaSki
Relative OD value
shNC
shNSUN2-1
shNSUN2-2
(hour)
CCK-8-SiHa
Relative OD value
shNC
shNSUN2-1
shNSUN2-2
(hour)
(D)
(E)
RTCA-CaSki
Relative cell counts
shNC
shNSUN2-1
shNSUN2-2
(hour)
RTCA-SiHa
Relative cell counts
shNC
shNSUN2-1
shNSUN2-2
(hour)
(F)
(G)
Cell Cycle-CaSki
Cell cycle distribution
G1
S
G2
shNC
shNSUN2-1
shNSUN2-2
Cell Cycle-SiHa
Cell cycle distribution
G1
S
G2
shNC
shNSUN2-1
shNSUN2-2

Supplementary Figure S3: (A) NSUN2 immunohistochemistry analysis of tissue chip consisting of 29 normal cervix and 30 cervical cancer paraffin sections. **(B, C)** Effect of NSUN2 knockdown on cell proliferation in CaSki **(B)** and SiHa **(C)** cells analyzed by CCK-8. **(D, E)** Effect of NSUN2 knockdown on cell proliferation in CaSki **(D)** and SiHa **(E)** cells analyzed by RTCA assay. **(F, G)** Effect of *NSUN2* knockdown on cell cycle distribution in CaSki **(F)** and SiHa cells **(G)** analyzed by flow cytometry analysis. (RTCA: real-time cell analysis; data shown is mean ± SEM, n = 3; student's unpaired t-test was used for statistical analysis.)

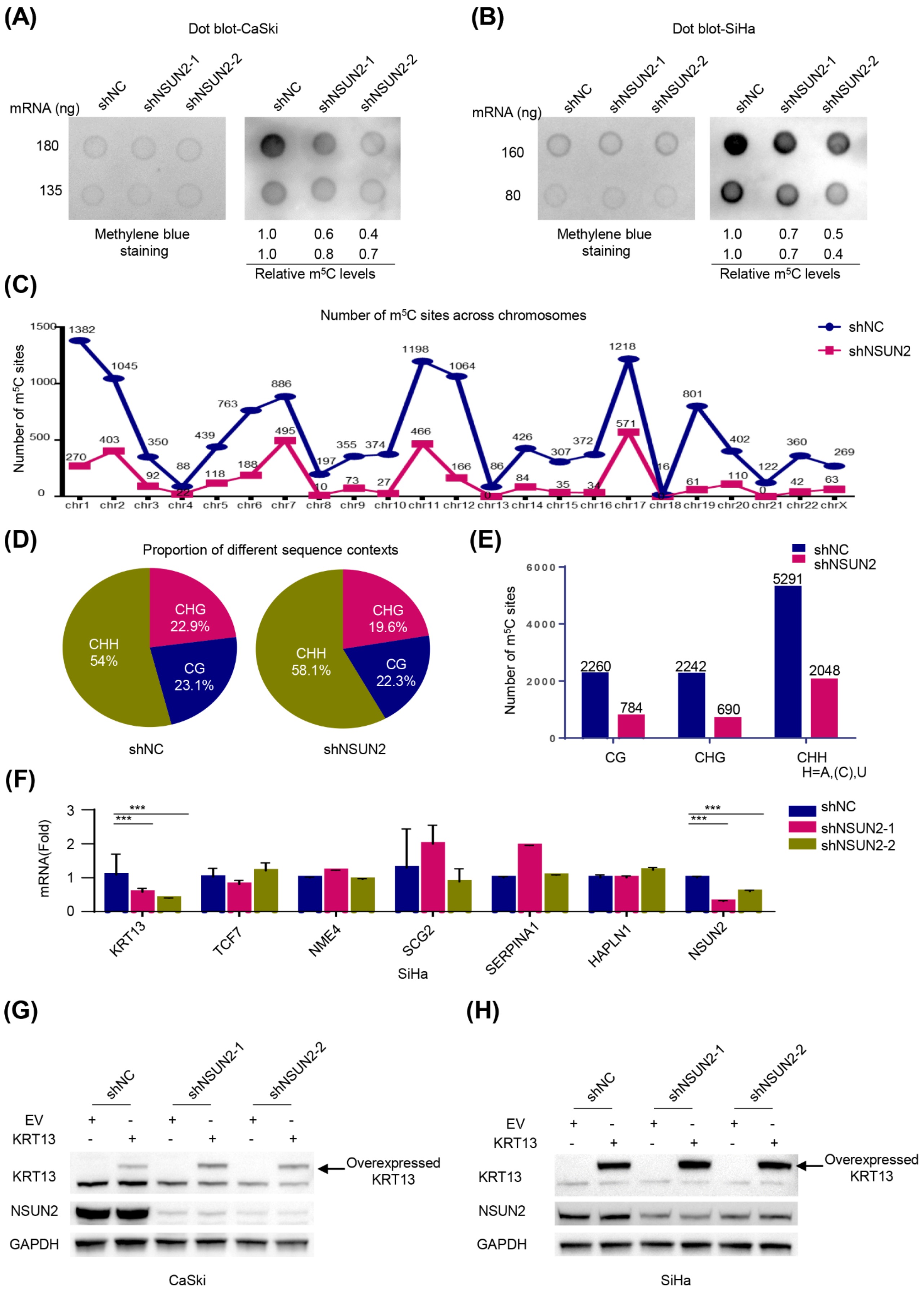
(A)
Dot blot-CaSki
mRNA (ng)
shNC
shNSUN2-1
shNSUN2-2
180
135
Methylene blue staining
1.0 0.6 0.4
1.0 0.8 0.7
Relative m5C levels
(B)
Dot blot-SiHa
mRNA (ng)
shNC
shNSUN2-1
shNSUN2-2
160
80
Methylene blue staining
1.0 0.7 0.5
1.0 0.7 0.4
Relative m5C levels
(C)
Number of m5C sites across chromosomes
Number of m5C sites
shNC
shNSUN2
chr1 chr2 chr3 chr4 chr5 chr6 chr7 chr8 chr9 chr10 chr11 chr12 chr13 chr14 chr15 chr16 chr17 chr18 chr19 chr20 chr21 chr22 chrX
(D)
Proportion of different sequence contexts
CHG 22.9%
CHH 54%
CG 23.1%
shNC
CHG 19.6%
CHH 58.1%
CG 22.3%
shNSUN2
(E)
shNC
shNSUN2
Number of m5C sites
2260 784
2242 690
5291 2048
CG
CHG
CHH
H=A,(C),U
(F)
mRNA(Fold)
shNC
shNSUN2-1
shNSUN2-2
KRT13
TCF7
NME4
SCG2
SERPINA1
HAPLN1
NSUN2
SiHa
(G)
shNC
shNSUN2-1
shNSUN2-2
EV
KRT13
KRT13
NSUN2
GAPDH
Overexpressed KRT13
CaSki
(H)
shNC
shNSUN2-1
shNSUN2-2
EV
KRT13
KRT13
NSUN2
GAPDH
Overexpressed KRT13
SiHa

Supplementary Figure S4: (A, B) Effect of NSUN2 stable knock down on global m^5C level in CaSki **(A)** and SiHa **(B)** cells. mRNA was extracted and subjected to m^5C mRNA dot blot assay to measure the global m^5C level. Methylene blue staining (left panel) was used as a loading control. Image J was used to quantify the relative m^5C level (normalized to shNC group). **(C)** RNA m^5C abundance along the chromosomes according to RNA-BisSeq. **(D)** Proportions of RNA m^5C sites identified in each sequence context according to RNA-BisSeq: CG, CHG and CHH, where H = A, C, or U. **(E)** Number of RNA m^5C sites identified in each sequence context according to RNA-BisSeq. **(F)** Verification of 6 possible targets of NSUN2 in NSUN2 stably knock down SiHa cells by RT-qPCR. **(G, H)** Transfect efficiency of KRT13 overexpression in CaSki **(G)** and SiHa cell lines **(H)** analyzed by western blot (mean ± SEM, n = 3, Student's unpaired t-test, *** $P < 0.001$.)

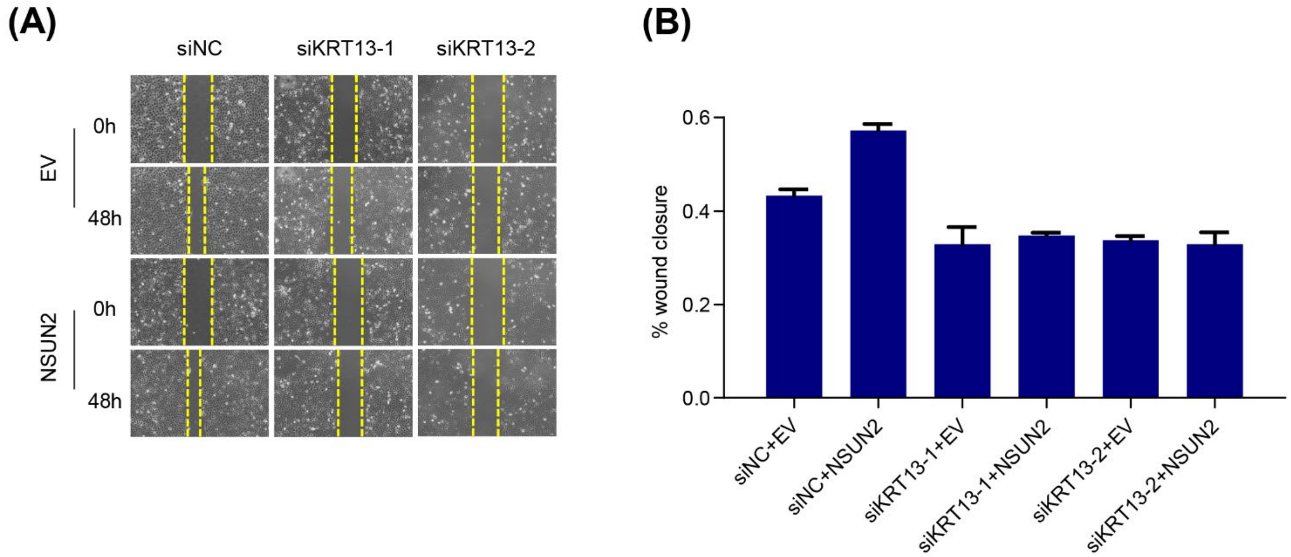

Supplementary Figure S5: (A, B) Effect of NSUN2 overexpression on cell migration in KRT13 knockdown CaSki cells as analyzed by wounding healing assay. Representative images (left panel) and quantification (right panel) of wounding healing assay showed the migration capability of CaSki cells. (EV: empty vector; data shown is mean ± SEM, n = 3.)

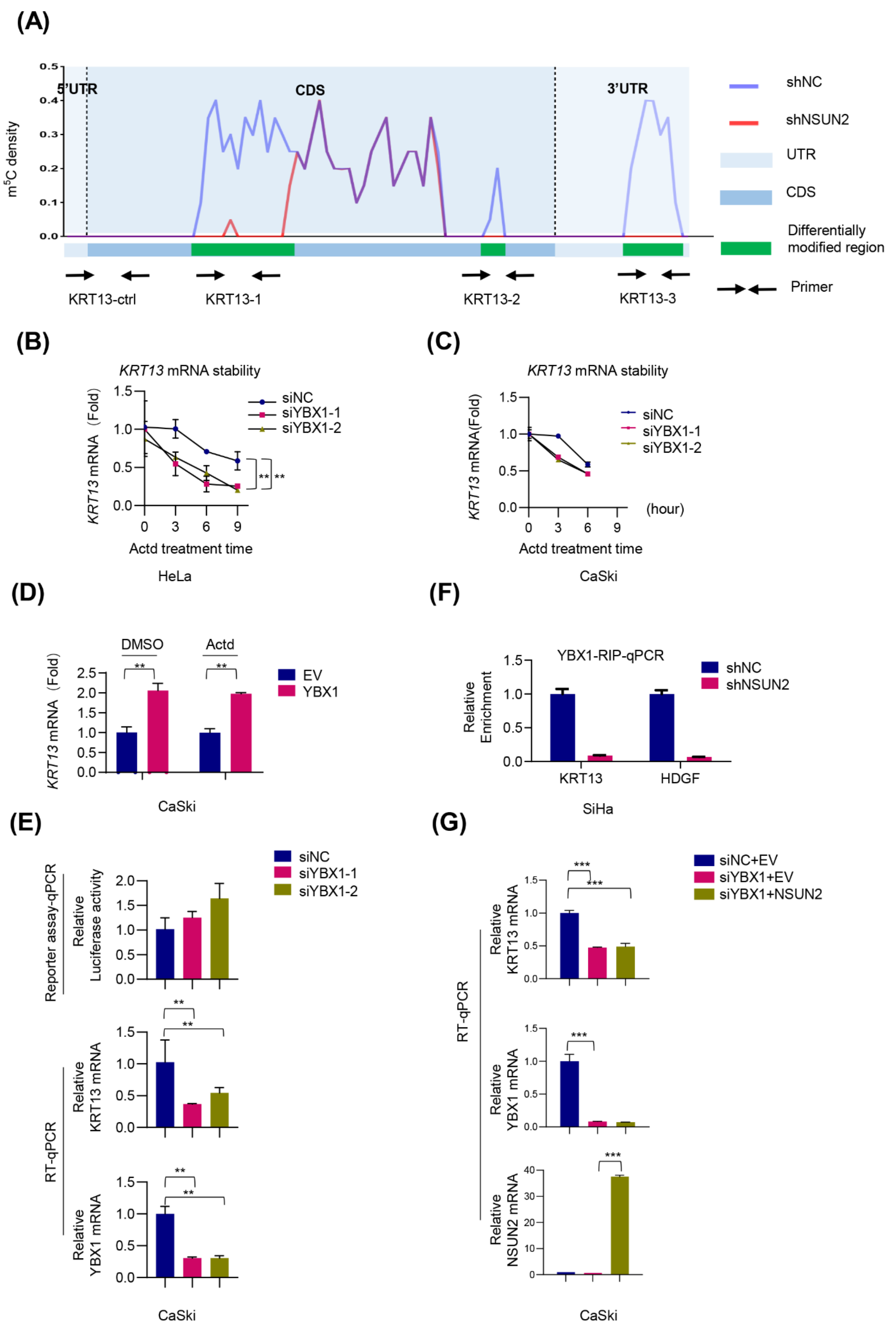
(A)
5'UTR
CDS
3'UTR
m5C density
0.5
0.4
0.3
0.2
0.1
0.0
shNC
shNSUN2
UTR
CDS
Differentially modified region
Primer
KRT13-ctrl
KRT13-1
KRT13-2
KRT13-3
(B)
KRT13 mRNA stability
KRT13 mRNA（Fold)
siNC
siYBX1-1
siYBX1-2
Actd treatment time
HeLa
(C)
KRT13 mRNA stability
KRT13 mRNA(Fold)
siNC
siYBX1-1
siYBX1-2
(hour)
Actd treatment time
CaSki
(D)
DMSO
Actd
KRT13 mRNA（Fold)
EV
YBX1
CaSki
(F)
YBX1-RIP-qPCR
Relative Enrichment
shNC
shNSUN2
KRT13
HDGF
SiHa
(E)
Reporter assay-qPCR
Relative Luciferase activity
siNC
siYBX1-1
siYBX1-2
RT-qPCR
Relative KRT13 mRNA
Relative YBX1 mRNA
CaSki
(G)
siNC+EV
siYBX1+EV
siYBX1+NSUN2
RT-qPCR
Relative KRT13 mRNA
Relative YBX1 mRNA
Relative NSUN2 mRNA
CaSki

Supplementary Figure S6: (A) Distribution density of m^5C modification on *KRT13* mRNAs in CaSki cells according to RNA-BisSeq data. Three specific pairs of primers were designed to target m^5C differentially modified region (*KRT13*-1,2,3) and another pair was designed as a negative control (*KRT13*-Ctrl). **(B, C)** Effect of YBX1 on the stability of *KRT13* mRNA. HeLa cells (B) or CaSki cells (C) transfected with two YBX1 siRNAs were treated with 10 µg/ml Actd for indicated time. Level of *KRT13* mRNA was analyzed by RT-qPCR. **(D)** CaSki cells overexpressing YBX1 were treated with DMSO or 10 µg/ml Actd for 3h. Level of *KRT13* mRNA was analyzed by RT-qPCR. **(E)** The promoter reporter assay showed the effect of YBX1 on the activity of KRT13 promoter. Lucifrase activity (upper pannel) in CaSki cells transfected with two YBX1 siRNAs was analyzed by RT-qPCR. **(F)** RIP-qPCR analysis of YBX1-*KRT13* interaction in NSUN2 knockdown and control SiHa cells. *HDGF* was used as positive control that can bind to YBX1 by m^5C. **(G)** Effect of NSUN2 in YBX1 knocked down CaSki cells. mRNA levels of KRT13, YBX1, NSUN2 in CaSki cell were analyzed by RT-qPCR. (EV: empty vector; data shown is mean ± SEM, $n = 3$; student's unpaired t-test was used for statistical analysis, *** $P < 0.001$.)

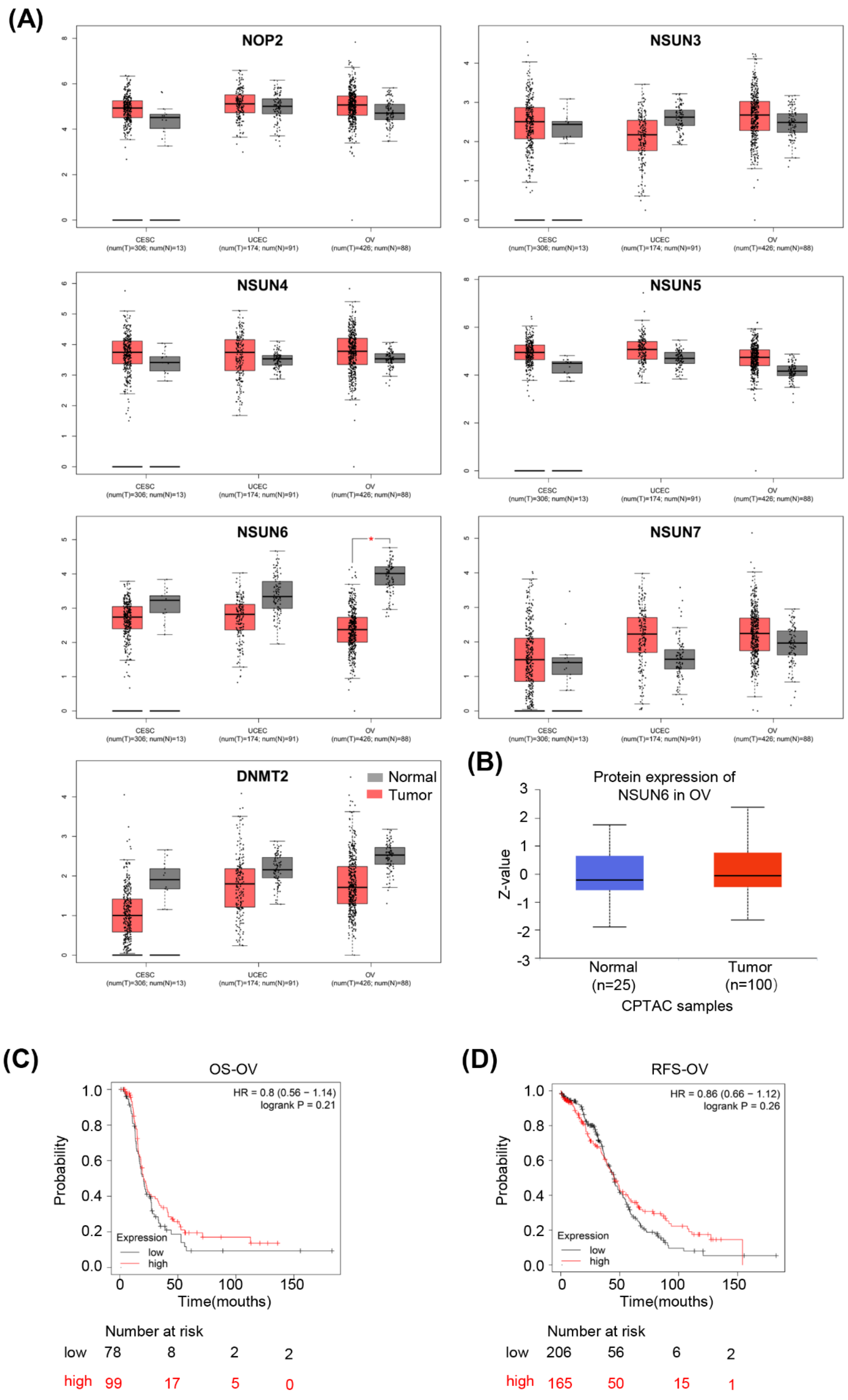
(A)
NOP2
NSUN3
NSUN4
NSUN5
NSUN6
NSUN7
DNMT2
Normal
Tumor
CESC
(num(T)=306; num(N)=13)
UCEC
(num(T)=174; num(N)=91)
OV
(num(T)=426; num(N)=88)
(B)
Protein expression of
NSUN6 in OV
Z-value
Normal
(n=25)
Tumor
(n=100)
CPTAC samples
(C)
OS-OV
HR = 0.8 (0.56 − 1.14)
logrank P = 0.21
Probability
Time(mouths)
Expression
low
high
Number at risk
low 78 8 2 2
high 99 17 5 0
(D)
RFS-OV
HR = 0.86 (0.66 − 1.12)
logrank P = 0.26
Probability
Time(mouths)
Expression
low
high
Number at risk
low 206 56 6 2
high 165 50 15 1

Supplementary Figure S7: (A) Box plot showed *NOP2, NSUN3, NSUN4, NSUN5, NSUN6, NSUN7, DNMT2* mRNA expression in cervical cancer, uterine cancer and ovarian cancer. GEPIA website was used to detect the mRNA expression of indicated proteins in three major gynecology cancers. **(B)** Box plot showed NSUN6 protein expression in ovarian tissues (normal vs. tumor) in CPTAC database assessed by UALCAN. **(C)** and **(D)** Association of NSUN6 mRNA expression with overall survival (OS) and recurrence-free survival (RFS) in RNA-Seq data in all ovarian cancer patients. (Data shown are mean ± SEM, n = 3, student's unpaired t-test was used for statistical analysis, * $P<0.05$. The log-rank test was used for Kaplan–Meier curves. The HR was performed using the Cox model.)

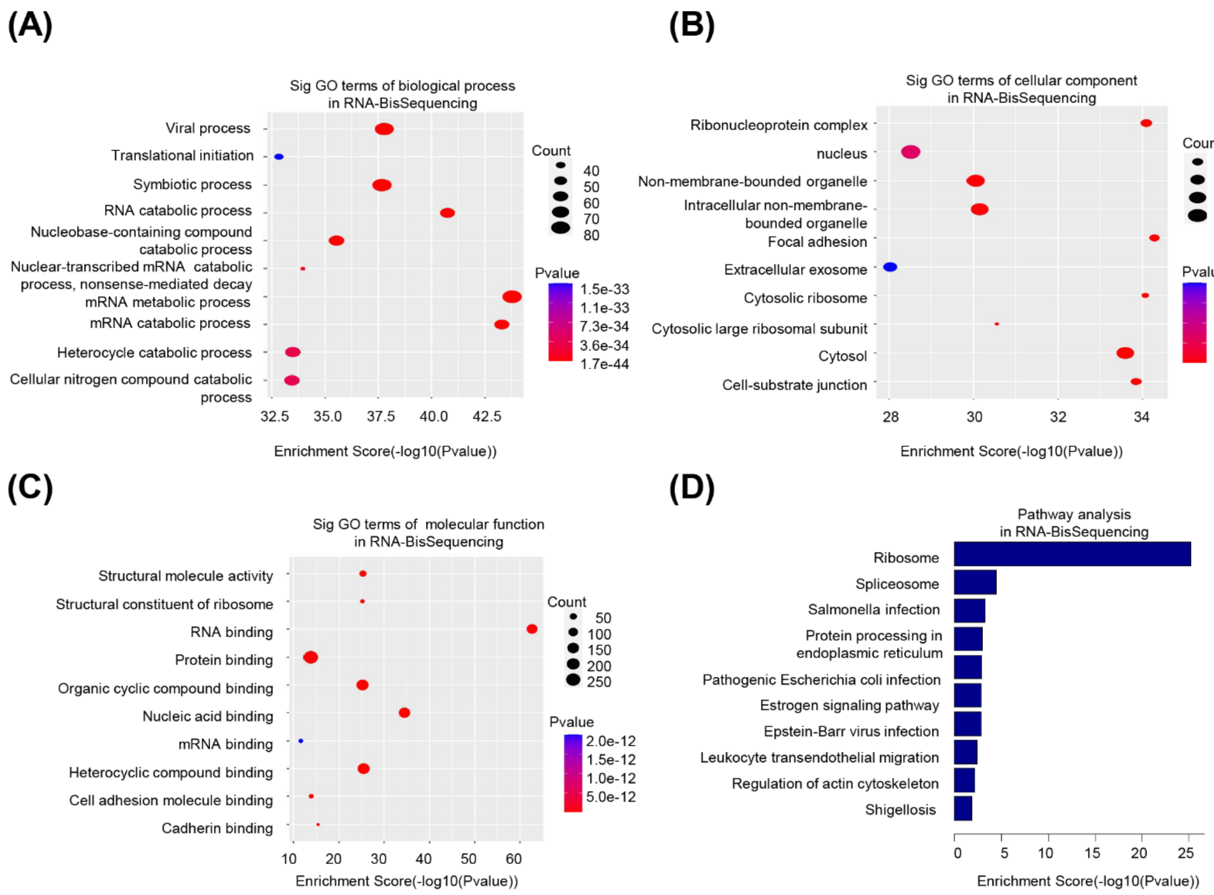

Supplementary Figure S8: (A) Top 10 significantly enriched biological processes of the differentially methylated genes in RNA-BisSeq results based on GO analysis. **(B)** Top 10 significantly enriched cellular component of the differentially methylated genes in RNA-BisSeq results based on GO analysis. **(C)** Top 10 significantly enriched molecular function of the differentially methylated genes in RNA-BisSeq results based on GO analysis. **(D)** Top 10 significantly enriched KEGG pathways of the differentially methylated genes in RNA-BisSeq results.
